# Why humans might help strangers

**DOI:** 10.3389/fnbeh.2015.00039

**Published:** 2015-02-20

**Authors:** Nichola J. Raihani, Redouan Bshary

**Affiliations:** ^1^Department of Genetics Evolution and Environment, University College LondonLondon, UK; ^2^Institut de Biologie, Université de NeuchâtelNeuchatel, Switzerland

**Keywords:** human cooperation, one-shot games, prisoner’s dilemma, error-management, cultural group selection, strong reciprocity

## Abstract

Humans regularly help strangers, even when interactions are apparently unobserved and unlikely to be repeated. Such situations have been simulated in the laboratory using anonymous one-shot games (e.g., prisoner’s dilemma) where the payoff matrices used make helping biologically altruistic. As in real-life, participants often cooperate in the lab in these one-shot games with non-relatives, despite that fact that helping is under negative selection under these circumstances. Two broad explanations for such behavior prevail. The “big mistake” or “mismatch” theorists argue that behavior is constrained by psychological mechanisms that evolved predominantly in the context of repeated interactions with known individuals. In contrast, the cultural group selection theorists posit that humans have been selected to cooperate in anonymous one-shot interactions due to strong between-group competition, which creates interdependence among in-group members. We present these two hypotheses before discussing alternative routes by which humans could increase their direct fitness by cooperating with strangers under natural conditions. In doing so, we explain why the standard lab games do not capture real-life in various important aspects. First, asymmetries in the cost of perceptual errors regarding the context of the interaction (one-shot vs. repeated; anonymous vs. public) might have selected for strategies that minimize the chance of making costly behavioral errors. Second, helping strangers might be a successful strategy for identifying other cooperative individuals in the population, where partner choice can turn strangers into interaction partners. Third, in contrast to the assumptions of the prisoner’s dilemma model, it is possible that benefits of cooperation follow a non-linear function of investment. Non-linear benefits result in negative frequency dependence even in one-shot games. Finally, in many real-world situations individuals are able to parcel investments such that a one-shot interaction is turned into a repeated game of many decisions.

## Human helpfulness

**Figure 1:**
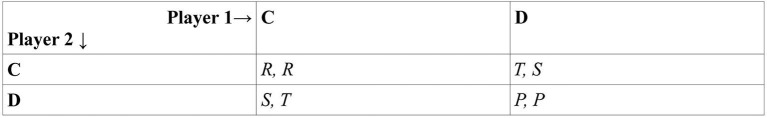
**Payoffs accruing to (Player 1, Player 2) according to each player’s decision to cooperate (C) or defect (D) in a social dilemma are shown. R is the reward for mutual cooperation, T is the temptation to defect, S is the sucker’s payoff and P is the punishment for mutual defection**. A game satisfies the assumptions of the prisoner’s dilemma where *T* > *R* > *P* > *S*. The snowdrift game is captured wherever *T* > *R* > *S* > *P*. Thus, the prisoner’s dilemma and the snowdrift game differ only in the best possible response to a partner’s defection: in the prisoner’s dilemma, the best response is to defect whereas in the snowdrift game, the best response is to cooperate.

One doesn’t have to observe humans in their natural habitat for long to witness many and varied examples of prosocial behavior, often directed towards complete strangers. People might vacate a seat on a crowded bus or train to let an elderly person sit down; hold open a door for others; or help a struggling parent to carry their pram down a flight of stairs. Humans also willingly donate resources, such as money or food, to others for example by giving to charity (e.g., Frey and Meier, 2004; Soetevent, 2005). These charitable donations are typically directed at recipients the helpful individual does not know or will expect to meet in the future. This propensity to help unrelated others who reside outside our regular social circle is striking when one considers that these helpful acts are seemingly unobserved and many of the interactions are unlikely to persist beyond the current round. To explain why humans are so helpful under conditions that seem to predict selfishness, researchers have attempted to bring this behavior into the laboratory by creating stylized games where subjects can cooperate with or help one another, but where payoffs are structured such that self-interested behaviors yield greater rewards. One of the most widely-used paradigms is the anonymous one-shot prisoner’s dilemma game (Luce and Raiffa, 1957). In this game, two interacting players simultaneously choose between cooperating or defecting. Payoffs are structured such that mutual cooperation yields higher payoffs for both than mutual defection, but defecting yields a higher individual payoff than cooperating regardless of how the partner behaves. Hence there is a temptation to defect even if the partner cooperates (see Figure 1). This game can be modeled with more than two players with similar incentives, yielding a temptation to defect when others cooperate: this is an n-player prisoner’s dilemma game (also commonly referred to as a public goods game, Ledyard, 1995). Regardless of whether the game is played with two or more than two players, in a one-shot prisoner’s dilemma the evolutionarily stable strategy (Maynard-Smith, 1982) is to defect. Another game that has been widely used is the dictator game (Kahneman et al., 1986). This is a two-player game where one player (the “dictator”) is endowed with a sum of money and can choose to give none, some or all of the endowment to the partner. The “receiver” in the game has no power and must accept any division of the endowment offered by the dictator. As in the prisoner’s dilemma game, individuals can maximize their earnings in the game by behaving selfishly and keeping the entire endowment.

Empirical studies have shown that humans often cooperate in anonymous one-shot prisoner’s dilemma games (or give money in dictator games) despite the fact that not contributing is the optimal solution (Camerer, 2003; Henrich et al., 2005; Engel, 2011). It has been suggested that helping in the absence of obvious rewards emerges from proximate psychological mechanisms that make helping others subjectively rewarding for the actor (Andreoni, 1990; Fehr and Camerer, 2007; Harbaugh et al., 2007). To explain why these psychological mechanisms exist, one has to ask under which conditions they are likely to have been favored by Natural Selection. The two dominant current explanations—the “big mistake hypothesis” (also known as the “mismatch hypothesis”) and the “cultural group selection hypothesis”—mainly disagree on the question of whether anonymous one-shot interactions were absent in human evolutionary history until very recently, and hence whether helping strangers is based on a psychological constraint or is instead adaptive under certain circumstances (specifically, when interaction partners belong to the same cultural group) (Rand and Nowak, 2013). Here, we first present these two hypotheses in more detail. We then move on to discuss how the laboratory game settings may differ from real-life interactions with strangers not only with respect to population structure but also in other fundamental ways. Based on this assessment, we will argue that there are several plausible routes by which cooperative behavior towards strangers could increase an individual’s direct fitness (Table 1). We hope that this evaluation will stimulate researchers to use or develop new experimental paradigms, such that our knowledge of the proximate mechanisms that underpin cooperation can be complemented with a better understanding of the adaptive significance of such strategies.

**Table 1:** **The key features of different theories to explain why humans cooperate in ostensibly anonymous, one-shot encounters**.

Theory	Where did strategies used in lab games evolve?	How might individuals benefit from helping strangers?	Is helping behavior adaptive in anonymous, one-shot lab games?
Mismatch/Big-mistake	Strategies evolved in context of repeated interactions with known partners.	No benefit	No
Between group competition	Strategies evolved to deal with (in-group) strangers even in one-shot, anonymous situations.	Benefit occurs in context of between-group competition. Unclear whether individual benefits directly (interdependence) or whether benefits accrue indirectly to kin residing in the group.	Maybe—if other people in the game are the relevant in-group and if helping in this setting somehow increases the group’s performance in competition with other groups. This prediction needs empirical verification.
Misperceiving interaction duration or anonymity	Strategies evolved to deal with strangers in situations with a probability of being anonymous/one-shot but where there was uncertainty over these parameters.	Individuals benefit from helping strangers if there are asymmetries in costs of misperceiving interaction duration/anonymity.	No
Partner choice via exploration	Strategies evolved to identify potential cooperative interaction partners from the population.	Individuals benefit from helping if this allows them to identify cooperative partners in the population and to establish relationships with these partners.	No
Alternative payoff matrices	Strategies evolved in the context of non-linear games.	Individual can benefit from helping if benefits of investing in public good are negative frequency dependent.	No
Parceled investments	Strategies evolved in contexts where individuals could parcel investments into smaller units, thereby turning one-shot interactions into games involving repeated decisions.	Individuals benefit if partner’s cooperative decisions/continuing the interaction are conditional upon the individual acting helpfully.	No

## Cooperation—a big mistake?

Some researchers have argued that the expression of prosocial behaviors in laboratory anonymous, one-shot encounters can be explained by mis-firing of psychological mechanisms in a context we are not evolutionarily adapted to (e.g., Cosmides and Tooby, 1989; Hoffman et al., 1998; Ben-Ner and Putterman, 2000; Johnson et al., 2003; Tooby et al., 2006). The line of reasoning is that the proximate mechanisms underpinning human social behavior evolved in close-knit, small groups comprised of kin and stable interaction partners, where no interaction was ever truly one-shot or anonymous. In such an environment, an individual that was predisposed to help group members would likely have been compensated for their investment, either via indirect benefits to kin (Hamilton, 1964a,b) or via personal benefits arising from future interactions with the recipient (Trivers, 1971) or observers (Nowak and Sigmund, 1998; Roberts, 1998). It is clear that these putative ancestral environments were fundamentally different to the modern day environment of most humans and especially to the artificial setup of most laboratory games. It is argued, therefore, that subjects in laboratory settings rely on psychological mechanisms that evolved in the context of repeated, non-anonymous interactions and that our predisposition to cooperate in the lab (or with strangers in the real world) might, therefore, be an evolutionary relic of times gone by. In support of the so-called (Boyd and Richerson, 2002; Fehr and Henrich, 2003) mismatch or big mistake hypothesis, there are several instances where imperfect human behavior can be attributed to the mismatch between our evolved psychology and our current environment. For example, the common tendency to be phobic of ancestrally dangerous stimuli (e.g., snakes, spiders) relative to more pressing modern-day threats (e.g., cars, guns); and the proclivity to ingest excessive quantities of foods that are high in sugar, fat and salt based on their relative abundance nowadays as compared with ancestral environments (Irons, 1998) can both be explained as psychological mechanisms that on average produced fitness enhancing outcomes in ancestral environments but that no longer deliver such benefits—and can even be harmful—in the modern world (Hagen and Hammerstein, 2006; Tooby et al., 2006).

Nevertheless, critics of the big mistake hypothesis have questioned the validity of assumption that ancestral social environments were devoid of anonymous one-shot encounters. Instead, it seems probable that even ancestral hunter-gatherers probably had some encounters with strangers with no obvious future follow-up, for example in the context of interactions outside their immediate social group (e.g., trading, feuds and so on, Lee, 1972; Wiessner, 1982; Keeley, 1996; Fehr and Henrich, 2003; Hill et al., 2011). It can be shown that, despite our evolutionary heritage, humans are able to identify interactions where helping is likely to yield a return benefit (e.g., by identifying kin, by predicting when behavior is likely to be observed or not, or when interactions are likely to be repeated)—and adjust behavior accordingly (Fehr and Henrich, 2003). For example, various studies have shown that people recognize and preferentially help kin over non-kin (Barber, 1994; Gurven, 2004); that we cooperate more when investments are public rather than anonymous (e.g., Milinski et al., 2002; Andreoni and Petrie, 2004; Soetevent, 2005; Lamba and Mace, 2010); and that we cooperate less when interactions will not be repeated or will end soon (e.g., Gächter and Falk, 2002; Gächter et al., 2008). Moreover, recent work has suggested that while people may use heuristics from daily life to guide cooperative decision making, these heuristics can be rapidly updated to reflect the conditions imposed in the artificial lab setting (Rand et al., 2012, 2014). In fact even fish are apparently able to adjust levels of cooperation to the likelihood of repeated interactions (Oates et al., 2010). Thus, critics of the big mistake hypothesis have good arguments that humans do have the cognitive machinery to detect when an interaction is likely to yield direct return benefits, and to adjust behavior accordingly.

If, as seems likely, interactions with strangers probably did exist in our evolutionary history, why do humans have a psychology that seems geared towards cooperating in these contexts, given that the short-term, payoff-maximizing approach is apparently to defect under such conditions? For cooperative investments to come under positive selection, the behavior must form part of a strategy that on average increases the fitness of the bearer. In other words, cooperative actors must somehow ultimately be repaid for their investments. Broadly, it has been suggested that the ultimate benefits associated with making costly investments could arise either with or without assortative population structure (often referred to as group-level (or multi-level) and individual-level selection in the literature). We consider these two arguments in turn.

## Between-group competition

Whenever populations are structured into groups of relatively stable composition, there is potential for competition between groups. The stronger such competition between groups is the more individual and group interests are closely aligned, and selection may favor strategies that prioritize unconditional contributions towards group success. The tendency for such “multi-level selection” to promote cooperation is seen at all levels of life and several of the major evolutionary transitions identified by Szathmáry and Smith (1995) rely on the repression of lower order conflict to bring about a higher-level advantage. According to accounts of helping based on between-group competition in humans (e.g., Gintis, 2000; Henrich, 2004; Boyd and Richerson, 2009), group-level benefits favor individual costly investments (even in the absence of obvious mechanisms to be directly compensated) because within-group helping generally predicts group success in the face of extinction threats (e.g., due to competition with other groups, environmental catastrophes etc., Gintis, 2000). Similar arguments emphasizing the importance of group-level benefits have been proposed by biologists for the evolution of helping behavior among non-relatives in cooperatively breeding groups (e.g., Kokko et al., 2001) and have been formulated with the notion of interdependence replacing the relatedness term in Hamilton’s rule (Roberts, 2005). Although group-level benefits could theoretically arise via genetic group selection, the high levels of between-group genetic variance that would be necessary to facilitate selection are thought to be unrealistic given the genetic evidence for (female) migration among groups (Seielstad et al., 1998; Bell et al., 2009). Moreover, since genetic between-group selection is thought to be a small force in large groups, it is unlikely to account for large-scale cooperation seen in modern day human societies. Thus, colleagues have focussed instead on the concept of cultural group selection, whereby immigrating individuals are expected to adopt the cultures of the new group, thereby reducing the behavioral variance that migrating individuals would otherwise have (Boyd and Richerson, 1982; Henrich, 2004; Bell et al., 2009). Cultural group selection of cooperative traits is thought to be facilitated by a general predisposition to learn socially from others, which would be adaptive where the cost of information acquisition was sufficiently high (Boyd and Richerson, 1982, 2009; Henrich and Boyd, 2001; Guzmán et al., 2007; Richerson and Boyd, 2008; but see Eriksson and Coultas, 2009; André and Morin, 2011). Selection for social learning outside the cooperative domain could then facilitate the evolution of cooperative cultural norms within populations. If populations varied in these norms and if the outcomes of competition between populations varied according to within-group levels of cooperation, then cultural group selection could favor cooperative strategies. The benefits of within-group cooperation could, in turn, select for psychological predispositions to cooperate with in-group members while punishing defectors (i.e., “strong reciprocity”, Gintis, 2000; Fehr et al., 2002; Fehr and Henrich, 2003; Boyd and Richerson, 2009), even in anonymous, one-shot encounters. Evidence for cross-cultural variation in cooperative tendency across societies (Henrich et al., 2001, 2005, 2006, 2010; Gächter and Herrmann, 2009; Gächter et al., 2010; House et al., 2013) has been touted as key evidence for the existence of between-group variation in cooperative norms that could be the basis for such selection (Henrich, 2004; Henrich et al., 2005).

Nevertheless, the assumptions underpinning the cultural group selection account of human cooperation have been subject to debate (Burnham and Johnson, 2005; Hagen and Hammerstein, 2006; West et al., 2011). For instance, while it is often assumed that the predisposition for conformity necessary to catalyze the emergence of within-group cooperation is an adaptive trait, it has been demonstrated that non-conformist transmission dominates conformist strategies in evolutionary models (Eriksson and Coultas, 2009). Moreover, analytical models have shown that a tendency for conformist transmission can undermine the evolution of helping behaviors (Feldman et al., 1985; Lehmann et al., 2008), not catalyze them as was originally predicted. Thus, in contrast to the predictions of the cultural group selection models, it is apparently easier for costly helping strategies to evolve under genetic systems of inheritance rather than via culture. Another central assumption of cultural group selection models is that individuals are constrained by a predisposition for conformity to blindly adopt the behaviors of others, even when strategic non-conformity would increase biological fitness (e.g., Gintis, 2003; Boyd and Richerson, 2009). The validity of this assumption is contested (Hagen and Hammerstein, 2006; André and Morin, 2011; El Mouden et al., 2014; Morin, 2014). Instead, it has been argued that the conditions under which cultural group selection would be expected to produce a maladaptive tendency to copy altruistic behavior are prohibitively restrictive (El Mouden et al., 2014; Morin, 2014) and, moreover, that antagonistic co-evolution should act to curb psychological predispositions to copy maladaptive actions (El Mouden et al., 2014). Indeed, strong empirical evidence suggests that people are sensitive to the benefits of imitation and do not copy blindly as supposed (Rendell et al., 2011; Morgan et al., 2012; Morin, 2014). More recently, it has also been suggested that asymmetries among individuals within groups can facilitate within-group cooperation when there is between-group competition, without having to resort to cultural transmission, reciprocity or punishment (Gavrilets and Fortunato, 2014). Specifically, when some individuals are able to benefit more than others from the production of a (within-group) public good (e.g., if they are dominant to others in the group), it pays for these individuals to invest more in the production of the public good, and this effect is particularly pronounced when groups are in competition with one another. Thus, population structure and between-group competition can facilitate within-group cooperation even in the absence of culturally transmitted tendencies to copy cooperative and punitive behaviors.

Empirical evidence for cultural group selection has also been called into question. While the existence of inter-cultural variation in cooperative norms (e.g., Henrich et al., 2001, 2005) seemed initially supportive of the predictions of the cultural group selection models, more recent empirical work has shown that substantial within-culture variation in cooperation exists that can be explained by local demography and ecology rather than culture (Lamba and Mace, 2011; Nettle et al., 2011; Schroeder et al., 2014). The extent of within-culture variation has been demonstrated to be comparable to that previously observed between cultures (Lamba and Mace, 2011) and, since many of the former studies sampled only one or a few populations per culture, it is argued that much of the measured variation that has been attributed to cultural differences may not in fact exceed within-group variation in these traits. Furthermore, cultural group selection models do not predict unconditional help towards any recipient but instead only towards members of the relevant in-group. Out-group members should not receive help and may in fact be harmed (“parochial altruism”, Bernhard et al., 2006). There is no a-priori reason for human subjects in laboratory one-shot games to assume that the co-players are members of the in-group, and so deserving of help. One might just as easily expect that subjects assume that co-players are out-group members, which would not predict costly cooperative behavior. Finally, it appears biologically questionable that in-group members are indeed unfamiliar with each other and that they can be reasonably certain that there will not be any future interactions; such a scenario would be much more likely with out-group members. Thus, even with a cultural group selection account of cooperation, human behavior in stylized laboratory games still remains a puzzle because we have to understand why co-players are apparently treated as in-group rather than out-group members (Burnham and Johnson, 2005).

## Direct benefits without between-group competition

The cultural group selection approach makes assumptions about population structure (grouping) and competition between these units without specifying whether a tendency to help strangers increases indirect fitness (through relatedness) or direct fitness (through interdependence). An alternative approach is to investigate conditions under which helping strangers may yield direct benefits in the absence of any specific population structure. One plausible suggestion is that human cooperation in one-shot games can be thought of as a strategy that minimizes costly error types (Burnham and Johnson, 2005; Yamagishi et al., 2007; Delton et al., 2011; Morin, 2014). Error management theory assumes that where there is uncertainty over the perceptual accuracy of the environment (such that all is not necessarily as it seems) and there are asymmetries in the costs of false-positive and false-negative error types, then evolution should favor strategies that minimize the costlier of the two error types (Johnson et al., 2013). It is important to bear in mind that Natural Selection is expected to favor strategies that on average increase the fitness of the bearer rather than to produce perfect behavior in every context. In other words, assessment errors mean that adaptive strategies will sometimes produce behavioral mistakes (West et al., 2011; Morin, 2014). For example, consider a meerkat who hears an alarm call indicating the likely, but only probabilistic, presence of a predator. In such a scenario there are likely to be asymmetric costs associated with erroneous behavioral responses. Responding to a false alarm call by fleeing incurs energetic and opportunity costs, while failing to flee when the threat is real incurs a far higher possible cost of being caught by a predator. Based on these asymmetric costs of behavioral errors, a strategy of “if hear alarm call, then flee” might be on average adaptive even if it produces several behavioral errors (i.e., fleeing in response to false alarms). In the context of interactions with strangers, it may well be the case that humans experience perceptual uncertainty over several features of the interaction, any or all of which may favor strategies that err on the side of caution by cooperating even when there is little ostensible benefit to doing so. The uncertainty could stem from inaccuracies in perceiving the likely duration of the interaction, whether the interaction is truly anonymous, or the payoff matrix of the interaction. We discuss how perceptual uncertainty in any of these features might select for broadly cooperative strategies below.

### Misperceiving interaction duration or anonymity

It has been argued that cooperation can be favored by evolution if there is even a small possibility that the interaction will be repeated and if this possibility is fundamentally unpredictable (Delton et al., 2011). Such a strategy could be adaptive even if it produces several behavioral errors (i.e., cooperating when no return benefits are possible). In a laboratory setting, an experimenter can exogenously impose the one-shot structure on the game (such that subjects can be certain that the interaction is not repeated) but this is unlike real life interactions with strangers, where we might often experience a degree of uncertainty about whether we might meet again. Using agent-based simulations, Delton et al. (2011) showed, using a mix of agents playing either always-defect (ALLD) or the conditionally cooperative strategy tit-for-tat (TFT), that cooperation can indeed be favored so long as there is a non-zero probability that the interaction might be repeated (and the partner is TFT rather than ALLD). In this setting, uncertainty over the number of interactions favors cooperation also in interactions that turn out to be one-shot. Cooperation is favored because mistaking a repeated interaction for one-shot (and therefore defecting in the first round of the game) incurred a greater cost than mistaking a one-shot interaction for repeated (and therefore erroneously cooperating). This is due to the unforgiving nature of TFT, whereby defecting in the first round of the game prompts the partner to defect in the next round and thus establishes mutual defection for the duration of the interaction. Strategies that take a chance on the interaction being repeated (and the partner playing TFT) by cooperating in the first round could instead establish mutually productive, cooperative relationships with TFT partners. In support of the idea, it has additionally been argued that due to autocorrelation of individual locations over time, interacting with an individual once leads to an increased probability of interacting with the same individual again in the future (Krasnow et al., 2013). Thus, by definition, meeting a partner once implies that the interaction will be repeated and that conditionally cooperative strategies will prevail.

Nevertheless, the generality of these findings have been challenged on the grounds that only interactions with unrepentant (ALLD) and unforgiving (TFT) strategies were explored (McNally and Tanner, 2011; Zefferman, 2014a,b). ALLD is unrepentant in the sense that it is committed to play defect in all rounds; it cannot change its behavior if the interaction turns out to be repeated. TFT is unforgiving in the sense that any defection by the partner will be immediately reciprocated with defection. By contrast, it was verbally argued that the importance of uncertainty for catalyzing one-shot cooperation might be substantially reduced if agents employed strategies that allowed for flexible responses, either an increased propensity to cooperate once the interaction extended beyond round one or a non-zero probability to forgive cheating partners in a repeated interaction (McNally and Tanner, 2011). This is because, with flexible strategies, the importance of cooperating in the first round would be reduced substantially since cooperation could still be established (or re-established) beyond round one (McNally and Tanner, 2011). Thus, the question of why individuals cooperate in ostensible one-shot interactions would remain unresolved. Indeed, the inclusion of repentant and forgiving strategies under the same conditions of uncertainty has subsequently been shown to vastly diminish the advantage to cooperating in (ostensible) one-shot interactions; in some cases actually reversing the direction of selection (Zefferman, 2014a). Zefferman (2014a) proposes that the failure of the model to predict one-shot cooperation when an arguably more realistic strategy set is considered emphasizes the importance of social learning of local cooperative norms (i.e., the cultural group selection approach) for explaining one-shot cooperation. Ultimately, empirical studies are likely to be important for understanding whether defection in an ostensible one-shot encounter precludes cooperation from being established (as predicted by Delton et al., 2011; Delton and Krasnow, 2014) or, instead, whether humans are more likely to forgive an interaction partner who starts by defecting but then switches to cooperation if the interaction continues.

While the Delton et al. (2011) model assumed that psychological responses to cooperate evolved in the context of directly reciprocal interactions, one also has to consider that under real world scenarios, actions might also be observed by uninvolved bystanders, who adjust their behavior towards the actor accordingly. For example, under indirect reciprocity models, helpful acts are reciprocated by third-parties rather than by recipients. Misperceiving that an interaction is unobserved by bystanders might carry similar costs to misperceiving the likely duration, in that erroneous defection incurs greater costs than erroneous cooperation in both scenarios. Thus, error-management might still play a role in sustaining cooperation but because individuals can never be certain that their actions are unobserved, rather than because they misperceive interaction duration. If there is even a slight possibility that actions will be observed—and if being seen as unhelpful carries greater costs than helping when no one is watching (as has been proposed in models of judgment bias, Rankin and Eggimann, 2009)—then selection might favor psychological mechanisms that make us behave as though we are observed most of the time. Empirical evidence suggests that reputation concerns have an important influence on prosocial tendency: people are typically more cooperative in public rather than anonymous laboratory games (e.g., Andreoni and Petrie, 2004; Lamba and Mace, 2010) and even exposing people to subtle cues of being watched (in the form of eye images) increases prosocial behavior under some circumstances (e.g., Haley and Fessler, 2005; Bateson et al., 2006; but see Fehr and Schneider, 2010; Raihani and Bshary, 2012 for failed replications). The presence of potential observers is made even more important when one considers that, via gossip, one’s positive or negative actions could be broadcast to several “observers”, who need not even have been present at the time of the event (e.g., Sommerfeld et al., 2007, 2008; Feinberg et al., 2014). Thus, an error-managing strategy might often cooperate—even when interactions seem to be anonymous—to minimize the reputation costs of not cooperating if the interaction turns out to be observed.

### Partner choice via exploration

Many of the games used to explore cooperation under laboratory settings impose a forced-play structure on subjects: players cannot choose who they want to interact with or to leave unproductive relationships (Axelrod and Hamilton, 1981). While cooperation can evolve under such circumstances in artificial simulations (e.g., via clustering or assortment of cooperators, Nowak and May, 1992; Fletcher and Doebeli, 2009; but see Hauert and Doebeli, 2004), network reciprocity based on spatial structure does not seem to support cooperation in empirical studies (Grujić et al., 2014). Indeed, assuming that players are constrained to use pure strategies and are forced to interact with one another is unlikely to reflect the conditions under which real-word relationships operate. Instead, individuals are typically able to choose interaction partners, and can choose to continue interactions with cooperative partners while terminating relationships that prove unproductive (e.g., Noë and Hammerstein, 1994; Baumard et al., 2013).

The possibility for partner choice might therefore favor unconditionally helpful strategies if being observed as helpful increases the chance of being chosen for future interactions. Importantly, the chooser need not make costly investments to reimburse the helper for their actions. Instead, simply being chosen for a mutually productive interaction (e.g., producing offspring, cohabiting) could compensate the helper for their initial investment (e.g., Bshary and Grutter, 2006; McNamara et al., 2008). According to the “competitive altruism” theory, competition for interaction partners occurs within a biological market (Noë and Hammerstein, 1994); and individuals who produce the strongest signals of quality (via helping) will be preferred as partners (Roberts, 1998; Lotem et al., 2003; Barclay, 2011). Empirical evidence suggests that competitive altruism might be an important mechanism underpinning human helping behavior: people choose interaction partners based on cooperative reputation (e.g., Barclay and Willer, 2007; Sylwester and Roberts, 2010, 2013) and avoid defectors (Rockenbach and Milinski, 2011), hence individuals compete with one another to advertise helpful actions (Raihani and Smith, in press).

The possibility for partner choice could also promote cooperation, even with unknown individuals in an anonymous setting, because individuals can use cooperative first moves to test the response of the partner and then decide whether to continue the interaction or not. Error-management might still play a role but, unlike the Delton et al. (2011) model, the cost of not cooperating would be that one misses out on the chance to have a mutually productive relationship with the partner, rather than that one is stuck in a mutually destructive relationship. It has been demonstrated that where there is extrinsically maintained variation in cooperative tendency within a population (maintained by differences in quality or ability to invest in the partner, McNamara and Leimar, 2010, or by mutation, immigration, recombination or epistasis, McNamara et al., 2004), then this variation could select for cooperative strategies because this is a way to identify whether the partner is also cooperative (McNamara et al., 2004). In such scenarios cooperation can evolve whenever the benefit of interacting with a cooperator outweighs the benefit of occasional exploitation. Variability in cooperativeness, together with a long lifespan during which to reap the benefits of a productive partnership, can then pave the way for the evolution of choosiness because, given sufficient variation in partner quality it can pay to leave a less cooperative partner in hope of finding a more cooperative individual next time (Sherratt and Roberts, 1998; McNamara et al., 2008). In many real-world scenarios, initial cooperative acts might often be low cost (in comparison to the potential benefits of establishing a mutually productive relationship) but investments could increase as the relationship becomes more established (e.g., Roberts and Sherratt, 1998). For example, while we do not routinely see people handing out $100 bills to strangers, low cost helpful acts, such as holding a door open or assisting with heavy bags, are relatively commonplace. These low cost investments are consistent with the idea that cooperation could be used as an exploratory strategy to strike up mutually productive relationships with other individuals in the population. Nowadays, modern technology may even allow us to develop long-distance relationships as a consequence of chance encounters where we help or are helped by others.

### Alternative payoff matrices

Most theoretical and laboratory studies of cooperation in humans have assumed a prisoner’s dilemma type payoff-matrix, where benefits scale linearly with investments. In such scenarios, the payoff-maximizing strategy in a one-shot game is to defect regardless of how the partner(s) behave. As a consequence, explanations based on assortment, repeated interactions or relatedness are typically invoked to account for the emergence and stability of cooperative behavior. The assumption that all social dilemmas have the structure of an n-player prisoner’s dilemma is, however, flawed (Kollock, 1998; Archetti and Scheuring, 2011). Alternative social dilemmas with different payoff matrices can yield evolutionarily stable cooperative strategies without having to invoke assortment, relatedness or repeated interactions (e.g., Doebeli and Hauert, 2005; Archetti, 2009a,b; Archetti and Scheuring, 2011). For example, consider the 2-player snowdrift game. This game describes two drivers traveling in opposite directions when they come across a snowdrift blocking the road. Neither driver can get home unless the road is cleared. Although each driver prefers the other to do the clearing, each would also rather clear the snowdrift himself than for the snowdrift to not be cleared at all. As a consequence, in the two-player snowdrift game, the best response to a cooperative partner is to defect, while the best response to a defecting partner is to cooperate. Thus, the strategic payoffs differ markedly from those in more frequently used prisoner’s dilemma, where defecting always yields the highest payoff (Doebeli and Hauert, 2005; Figure 1). An n-player snowdrift game is often referred to as a volunteer’s dilemma (Diekmann, 1985). In its simplest form, this game assumes that a public good will be produced if one player cooperates and that additional investments do not increase the magnitude of the public good. Thus, unlike the traditional n-player prisoner’s dilemma, benefits are a non-linear function of investment and cooperation is therefore expected to be under negatively frequency dependent selection. As in the snowdrift game, the benefit of the public good being produced is larger than the cost associated with producing it, such that all players would do best to invest to produce the public good if no one else does so.

Relaxing the assumption of linearity has far-reaching consequences for the emergence and stability of cooperative strategies in n-player games. Specifically, when individuals are unsure about how others are likely to behave in a non-linear public goods game, then the best strategy is to cooperate probabilistically (where the probability depends on the cost to benefit ratio of cooperating and group size, Archetti, 2009a,b; Archetti and Scheuring, 2011, 2012). Such probabilistic strategies will emerge even in non-repeated games without spatial assortment or interactions among relatives. Where players differ in their ability to invest, or in the benefit that they can extract from the public good being produced, then this can also offer a potential solution to a non-linear public goods game, with those players who will reap the largest benefit from investing being more likely to contribute to the public good (e.g., see Gavrilets and Fortunato, 2014; Szolnoki and Perc, 2014). With respect to the assumption of linearity in benefits, n-player prisoner’s dilemma games lie at one end of a spectrum with threshold Public Goods Games (i.e., volunteer’s dilemmas) at the other end. All intermediate cases (where benefits are a sigmoidal function of investment in the public good) resemble the volunteer’s dilemma more than the traditional n-player prisoner’s dilemma in that they also yield a stable mixed equilibrium of cooperators and defectors in the population, even in the absence of other incentives to cooperate (Archetti and Scheuring, 2011). Thus, it has been argued that many biological examples of cooperation in social dilemmas are more likely to yield non-linear rather than linear benefits, which has profound implications for our understanding of how cooperation evolves and is maintained in these scenarios (Kummerli et al., 2007; Archetti, 2009a; Sherratt et al., 2009; Archetti et al., 2011; Archetti and Scheuring, 2012). For example, the costly production of invertase in yeast, alarm calling in animal groups and the formation of fruiting bodies in social amoebas are all examples that can be described as non-linear public goods games where cooperation is under negative frequency dependent selection (Gore et al., 2009; Archetti and Scheuring, 2011; Archetti et al., 2011). Yeast growth requires the costly production of the enzyme invertase to hydrolyze sucrose into smaller glucose molecules which can be imported into the cell (Gore et al., 2009). Although invertase production is costly and can be parasitized by non-producing cells, a complete lack of invertase can be lethal, meaning that producer cells outperform non-producers when rare. Conversely, at high densities of producers, non-producing cells have an advantage because they can parasitize the invertase being produced by the other cells (Gore et al., 2009).

For humans, it is less clear whether the majority of the social dilemmas that have shaped our social behavior ought to be described with linear or with non-linear payoff functions. In the case of punishment, which has been modeled as a second-order public good (and often therefore explained in terms of cultural group selection, e.g., Boyd et al., 2003), it has been argued that the payoffs of investing in punishment (in terms of increased within-group cooperation) are likely to scale non-linearly with number of punishers, thereby providing a direct individual-level solution for its existence (Raihani and Bshary, 2011). Other social dilemmas that have been explained in terms of cultural group selection, for example contributions to group defense during war, might also be more likely to have non-linear than linear payoffs. Group survival, which is the typical currency for payoffs associated with cooperating in warfare, is likely to be a non-linear function of contributions to defense, meaning that the payoffs associated with increasing within-group cooperation are by definition non-linear. It may turn out to be the case that linear public goods problems exist mainly in artificial laboratory settings and that subjects use strategies and psychology that evolved predominantly in the context of non-linear games when they participate. Specifically, if most real-world public goods problems are non-linear in nature and if there is always a certain degree of uncertainty about whether others will contribute to produce the public good (thereby obviating the need for the subject’s own investment), selection may have favored strategies that either probabilistically cooperate (when cooperation is binary) or that invest intermediate amounts (when cooperation is a continuous variable) (c.f. Kummerli et al., 2010), even in one-shot games.

### Parceled investments

Finally, we would like to highlight an additional discrepancy with the way laboratory prisoner’s dilemma experiments are set up compared with how interactions typically occur in the real-world. In many experimental games, the act to cooperate or to defect is an all-or-nothing action where players press a button, and typically learn about each other’s choices *post hoc*. On the other hand, interactions with strangers in our evolutionary past (e.g., in the context of trades) are highly unlikely to have involved exchange of closed boxes where players only found out after separating what the other put in the box. Instead, real-world interactions with strangers might often have involved simultaneous or parceled exchanges, where individuals could monitor the behavior of one another, make behavioral adjustments in real time and—importantly—terminate unproductive exchanges prematurely (e.g., Connor, 1992; Hart and Hart, 1992). For example, most female lions approach intruders simulated by playbacks more slowly if they teamed up with laggard female group members, apparently looking back regularly to check the spatial configuration of self vs. partners (Heinsohn and Packer, 1995). Theoretical models have shown that where cooperation is not an all-or-nothing event but investments can instead can be parceled and adjusted in real time, then prisoner’s dilemma type situations can be solved cooperatively, even in one-shot anonymous games (Friedman and Hammerstein, 1991; Connor, 1995; Johnstone and Bshary, 2002). The key issue is that stable cooperation does not rely on repeated interactions but on repeated decisions. The question of whether encounters between strangers (either currently, or in our evolutionary past) are more likely to involve single vs. multiple decisions remains open for empirical exploration.

## Conclusion

We have discussed potential explanations for the observation that humans help complete strangers under natural conditions and often cooperate in laboratory anonymous one-shot games. While the extent to which humans encountered such situations in our evolutionary past necessarily remains an open debate, it is clear that such encounters happen today. Moreover, it appears that we are at least partly adapted to adjust behavior to such situations. Our aim was to identify functional explanations for why humans regularly help strangers under natural conditions. The main take-home message is that the laboratory settings deviate from natural encounters in various important ways that make helping in real-world encounters potentially under positive selection while it is clearly not in the lab setting. Error-management arguments suggest that during natural encounters it is very hard to assess whether an interaction will be one-off and/or whether the interaction will remain truly anonymous. Furthermore, while lab games typically impose a forced-play scenario on subjects, in real-life individuals can choose to pursue productive relationships and abandon unproductive partners. This possibility for partner choice might select for helping behavior even with unknown strangers. Another issue is that in real-world interactions, the payoffs might often be a non-linear function of total investment, causing helping to be under negative frequency dependent selection rather than being altruistic. Finally, as soon as interactions involve multiple decisions, stable cooperation may be achieved even between strangers without any future perspective. We note that these different explanations are not mutually exclusive and that different explanations are likely to apply to different real-world scenarios. One way to explore the importance of the different explanations we proposed would be to use a wider variety of laboratory games, that replicate conditions that are likely to prevail in real-world interactions with strangers.

## Conflict of interest statement

The authors declare that the research was conducted in the absence of any commercial or financial relationships that could be construed as a potential conflict of interest.
